# Empirical investigation of the ethical reasoning of physicians and molecular biologists – the importance of the four principles of biomedical ethics

**DOI:** 10.1186/1747-5341-2-23

**Published:** 2007-10-25

**Authors:** Mette Ebbesen, Birthe D Pedersen

**Affiliations:** 1 Centre for Bioethics and Nanoethics, University of Aarhus, Aarhus C, Denmark; 2 Visiting Researcher at the Kennedy Institute of Ethics, Georgetown University, Washington DC, USA; 3 Department of Nursing Science, University of Aarhus, Aarhus C, Denmark; 4 Faculty of Health Sciences, University of Aarhus, Aarhus C, Denmark

## Abstract

**Background:**

This study presents an empirical investigation of the ethical reasoning and ethical issues at stake in the daily work of physicians and molecular biologists in Denmark. The aim of this study was to test empirically whether there is a difference in ethical considerations and principles between Danish physicians and Danish molecular biologists, and whether the bioethical principles of the American bioethicists Tom L. Beauchamp and James F. Childress are applicable to these groups.

**Method:**

This study is based on 12 semi-structured interviews with three groups of respondents: a group of oncology physicians working in a clinic at a public hospital and two groups of molecular biologists conducting basic research, one group employed at a public university and the other in a private biopharmaceutical company.

**Results:**

In this sample, the authors found that oncology physicians and molecular biologists employed in a private biopharmaceutical company have the specific principle of beneficence in mind in their daily work. Both groups are motivated to help sick patients. According to the study, molecular biologists explicitly consider nonmaleficence in relation to the environment, the researchers' own health, and animal models; and only implicitly in relation to patients or human subjects. In contrast, considerations of nonmaleficence by oncology physicians relate to patients or human subjects. Physicians and molecular biologists both consider the principle of respect for autonomy as a negative obligation in the sense that informed consent of patients should be respected. However, in contrast to molecular biologists, physicians experience the principle of respect for autonomy as a positive obligation as the physician, in dialogue with the patient, offers a medical prognosis based upon the patients wishes and ideas, mutual understanding, and respect. Finally, this study discloses utilitarian characteristics in the overall conception of justice as conceived by oncology physicians and molecular biologists from the private biopharmaceutical company. Molecular biologists employed at a public university are, in this study, concerned with allocation, however, they do not propose a specific theory of justice.

**Conclusion:**

This study demonstrates that each of the four bioethical principles of the American bioethicists Tom L. Beauchamp & James F. Childress – respect for autonomy, beneficence, nonmaleficence and justice – are reflected in the daily work of physicians and molecular biologists in Denmark. Consequently, these principles are applicable in the Danish biomedical setting.

## Introduction

### Developing a suitable method

The basic approach of biomedical ethics has primarily been philosophical inquiry with the aim of logical reasoning, conceptual clarity, coherence and rational justification [1]. Although such theoretical reflections make important contributions to the field, empirical researchers regard some of these attempts as remote from biomedical practice [2]. On the other hand, published empirical research on the ethical reasoning of nurses and physicians offers only descriptions of such reasoning. For instance, Lindseth & Norberg [3] developed a phenomenological hermeneutical method to reveal the morals and the ethical thinking of physicians and nurses based on interviews. According to Lindseth & Norberg [3] this method can be used to elucidate the essential understandable meaning of good and bad as actually lived in human experience. The method was inspired by Husserl's descriptive phenomenology in as much as the aim is to describe the lived experience of the interviewees. It is essential that the researcher has a phenomenological attitude, and sheds all prior personal knowledge to grasp the essential lived experience of the respondents [4]. Furthermore, it is important that the respondents shift to the phenomenological attitude, i.e. refrain from making judgements about the factual. According to Lindseth & Norberg [3]: "The easiest and, so to speak, the natural way of doing this is to narrate from lived experience". The approach is hermeneutical since the task is to understand the experiences expressed in the interview texts. Hermeneutics goes beyond the description of core concepts and essences to look for meanings embedded in life practices. These meanings are not always apparent to the respondents, but can be gleaned from the narratives (the interview texts) they produce [4]. Udén et al. [5] conducted a study using the phenomenological hermeneutical method of Lindseth & Norberg [3] to investigate the reflections of nurses and physicians in their narratives about ethically difficult care episodes. Udén et al. [5] concluded that the ethical thinking of nurses appears to be related to the ethics of care, whereas the ethical thinking of physicians is related to the ethics of justice. Moreover, the study [6] shows that nurses tell their stories within a relationship ethics perspective and that physicians tell their stories within an action ethics perspective. It remains unclear, however, whether nurses *ought *to assume a care or relationship ethics perspective, or if physicians *ought *to take a justice or action ethics perspective. Can the descriptive conclusions of the study have any normative implications for nurses and physicians? For instance, if an empirical study concludes that physicians adhere to a paternalistic doctor-patient relationship in which physicians do not respect the autonomy of the patient, does such a study then imply that physicians *ought *to adhere to such a paternalistic relationship? So the question remains whether there is any relationship between empirical findings and ethical theory about what *principles *(appendix, note 1) we ought to act in accordance with.

The results of the phenomenological hermeneutical method of Lindseth & Norberg [3] are descriptive in as much as they describe the lived experience of the respondents. In Ebbesen & Pedersen [7] we argue that the phenomenological hermeneutical method can be combined with the moral philosophical method of Wide Reflective Equilibrium (WRE) as a decision procedure for the formulation of normative principles, because WRE is a method or process of deciding what we should think, not merely one of describing what we do think [8]. So to achieve a normative approach, we combine the phenomenological hermeneutical method with the method of WRE.

The method of WRE is based upon the American philosopher John Rawls' theory for developing and justifying principles for a just society. Rawls speaks of a system with three levels: particular moral judgements, first principles, and general convictions. He claims that particular moral judgements are justified by the overall *coherence *(appendix, note 2) of the system and uses the term WRE to describe this state [9]. To achieve WRE, we start with our initial moral judgments. We begin by screening our initial moral judgements to eliminate those in which we have little confidence and those made under circumstances conducive to error. We then search for general moral principles that best account for the remaining considered moral judgements. We may find reason to revise or discard some of our considered moral judgments that conflict with highly plausible moral principles. Rawls imagines that the process of comparing principles with our considered judgments will lead us to go back and forth, sometimes modifying our principles and sometimes our considered moral judgements until the principles match, fit, or are in line with our considered moral judgements and consistency is achieved. Finally, we have to subject the moral principles we arrive at to alternative moral perspectives and the force of various arguments for these. WRE is achieved when our considered judgements match, or are in line with our general principles duly pruned and adjusted. However, this WRE is not necessarily stable. For instance, it may be liable to be upset by particular cases which lead us to revise our judgments or principles [9,10]. Moreover, the notions of 'match', 'fit', 'in line with' and 'consistency' are not well-defined. We understand the terms as meaning that WRE requires logical consistency between considered moral judgements and moral principles. Rawls writes that the justification of ethical principles "is a matter of the mutual support of many considerations, of everything fitting together into one coherent view" [10]. Rawls believes that if reasonable principles exist for deciding moral questions "there is a presumption that the principles of a satisfactory explication of the total range of the considered judgments of *competent judges *(appendix, note 3) will at least approximate them" [11].

In the light of an interpretation of the method of WRE as a decision procedure, the purpose of this empirical study is to validate, formulate and justify reasonable moral principles in specific biomedical practice. To make the approach normative, the interview guide was constructed in accordance with the theory of WRE so that the respondents could achieve WRE. For more details, please see Ebbesen & Pedersen [7]. By combining the phenomenological hermeneutical method with WRE as a decision procedure, we have an approach to bioethics where empirical research is integrated into the formulation of normative ethical principles, which means that the conclusions of this empirical study may provide health care professionals and biomedical researchers with normative principles about how to analyse, reason and act in practice in ethically difficult situations.

### Studying ethical reasoning of physicians and molecular biologists

Most qualitative empirical research in the field of biomedical ethics is concerned with the ethical reasoning of physicians and nurses [5,6,12-15]. Some researchers have been especially interested in studying differences in ethical reasoning between physicians and nurses, and between men and women [5,6,13]. Others have specifically investigated how physicians handle situations in which there is tension between the obligation to respect the patients' right to autonomy and the obligation to promote their health [14,15]. However, little is known about the differences in the ethical considerations at stake between physicians working in the clinical situation and molecular biologists conducting basic research.

This article presents partial findings from the larger research project 'Bioethics in Theory and Practice', in which our overall aim was to investigate the ethical reasoning of physicians and molecular biologists. We have three groups of respondents: a group of oncology physicians working in a clinic at a public hospital and two groups of molecular biologists conducting basic research, one employed at a public university and the other in a private biopharmaceutical company. The reason for selecting these three groups of respondents is to explore whether ethical problems about human beings are perceived as more distant for molecular biologists than for physicians due to the fact that physicians work with human beings in a doctor-patient relationship, whereas molecular biologists investigate human material such as DNA and cells in cultures. Another consideration might be the location of employment for molecular biologists, e.g. at a public university or in a private biopharmaceutical company. This difference in location of employment could influence the motive for ethical evaluation of research. Hypothetically, the motive for the ethical evaluation of the research of molecular biologists employed at a public university could be an interest in ethically good behaviour, in contrast to perhaps an overall economic motive for molecular biologists employed in a private biopharmaceutical company.

The findings of this larger project, however, are so comprehensive that they are being presented in several articles. The findings presented in other articles are summarised here.

We found similarities in the character of the daily work of molecular biologists employed in a private biopharmaceutical company and oncology physicians working in a clinic at a public hospital. With regard to research goals and driving force, both of these groups have a specific effort of beneficence in mind. Their motivation is to help sick people. Molecular biologists employed in a private biopharmaceutical company conduct basic research with the aim of developing pharmaceuticals. Oncology physicians treat patients suffering from serious cancer and perform clinical trials with the aim of developing cancer therapies. In contrast, molecular biologists employed at a public university do not have any specific effort of beneficence in mind in their daily work; their research goal is to provide answers to basic research questions.

With regard to funding and efficiency, we see similarities between the two groups of respondents employed by public institutions. Molecular biologists employed at a public university and oncology physicians employed at a public hospital think that a lot of time is spent on paperwork and that resources are limited. Both of these groups are under stress, either because they are applying for funding for basic research or because they are treating as many patients as possible per day in the out-patients' clinic. In contrast, molecular biologists employed in a private biopharmaceutical company have time allocated to read scientific articles and experience being able to decide the amount of work they will do themselves.

Oncology physicians working in a clinic at a public hospital perceive ethical evaluation as part of their daily work. They discuss in groups how to treat patients, and they have interdisciplinary seminars. This might be due to the fact that they work with human beings in a doctor-patient relationship in contrast to conducting basic research. On the other hand, molecular biologists employed at the university conducting basic research with the aim of answering basic research questions do not talk about ethics in their daily work and they do not want to prioritise participation in seminars on ethics because they have a busy schedule. This contrasts with the group of molecular biologists employed in a private biopharmaceutical company. The private company prioritises ethical evaluation because of the investors. If the company behaves unethically, they will be punished by the consumers and by the investors in the long run.

## Background

Researchers have particularly focused on formulating ethical principles that reflect the ethical issues at stake in biomedicine and on analysing what principles should be used to evaluate ethical problems in the field. After examining considered moral judgements in biomedicine, two American bioethicists, Tom L. Beauchamp & James F. Childress [16], became convinced that the principles of respect for autonomy, nonmaleficence, beneficence and justice are central to and play a vital role in biomedicine. In table 1, we present a brief formulation of the bioethical principles of Beauchamp & Childress.

**Table 1:** The Four Principles of Biomedical Ethics.  A brief formulation of the four bioethical principles of respect for autonomy, beneficence, nonmaleficence and justice of Beauchamp & Childress [16].

*The Principle of Respect for Autonomy*
• As a negative obligation: Autonomous actions should not be subjected to controlling constraints by others
• As a positive obligation: This principle requires respectful treatment in disclosing information, probing for and ensuring understanding and voluntariness, and fostering autonomous decision-making [16].
*The Principle of Beneficence*
• One ought to prevent and remove evil or harm
• One ought to do and promote good
• One ought to weigh and balance the possible goods against the possible harms of an action [16, 17].
*The Principle of Nonmaleficence*
One ought not to inflict evil or harm. Or more specifically: One ought not to hurt other people mentally or physically [16].
*The Principle of Justice*
Beauchamp hildress examine several philosophical theories of justice, including egalitarian theories which emphasise "equal access to the goods in life that every rational person values (often invoking material criteria of need and equality)" [16]. Beauchamp & Childress propose that "society recognize an enforceable right to a decent minimum of health care within a framework for allocation that incorporates both utilitarian and egalitarian standards" [16]. (Utilitarian theories emphasise "a mixture of criteria for the purpose of maximizing public utility") [16].

According to Beauchamp & Childress, no one principle ranks higher than the others. Which principles should be given most weight depends on the context of the given situation. Beauchamp & Childress consider the four principles as *prima facie binding*, i.e. they must be fulfilled, unless they conflict on a particular occasion with an equal or stronger principle. This type of principle is always binding unless a competing moral obligation overrides or outweighs it in a particular circumstance. Beauchamp & Childress write: "Some acts are at once prima facie wrong and prima facie right, because two or more norms conflict in the circumstances. Agents must then determine what they ought to do by finding an actual or overriding (in contrast to prima facie) obligation" [16]. This means the agents must locate the best balance of right and wrong by determining their actual obligations in such situations by examining the respective weights of the competing prima facie obligations (the relative weights of all competing prima facie norms). In the latest edition of their book, *Principles of Biomedical Ethics*, Beauchamp & Childress specify conditions that should be fulfilled for one prima facie principle to weigh heavier than another. They also describe how to specify the principles [16]. According to Beauchamp & Childress, there is no straightforward movement from principles to particular judgments. Principles are only starting points and, as such, general guidelines for the development of norms of appropriate conduct. Principles need to be supplemented by paradigm cases of right action, empirical data, organisational experience, etc. Rights, virtues and emotional responses are as important as principles for ethical judgement [16].

Beauchamp & Childress believe that the principles of their theory find support across different cultures. They claim that the principles are part of a cross-cultural common morality and that in all cultures people who are serious about moral conduct accept the norms of this common morality [16]. However, even though these principles are generally acknowledged, this does not mean that there is consensus about what is good and bad. Interesting debates occur when the principles are to be interpreted, specified and balanced in specific historical, social, economic and political contexts.

Although Beauchamp & Childress' theory is one of the most influential bioethical theories, it is, of course, also subject to much philosophical discussion [18-31]. Much of the criticism focuses on the application of principles, i.e. the problems of balancing and specification. However, some criticism is also focused on the choice of principles and their content. There is no doubt that Beauchamp & Childress were much inspired by W. D. Ross [32], who claims in *The Right and the Good *(1930) that common sense tells that we have some prima facie duties (also called conditional duties) to do special acts (e.g. keeping a promise). Ross [32] regards the duties of fidelity, reparation, gratitude, justice, beneficence, self-improvement and nonmaleficence as prima facie duties. In line with Ross, Beauchamp & Childress regard the four bioethical principles as prima facie binding and their choice of principles is almost in agreement with Ross' prima facie duties.

There is a lot of controversy about what principles reflect the ethical considerations at stake in biomedicine and, as a result, which principles should be used to analyse ethical problems in the field. Beauchamp [31] claims that the efficacy of the four principles can be tested empirically and that it can be determined whether they are part of a cross-cultural common morality. He does not present any empirical data generated systematically by qualitative research methods to support this position. However, he does invite the design of an empirical research project to investigate the question. What is needed is to construct an empirical study to investigate ethical reasoning in biomedicine. This was the overall purpose of the larger research project, 'Bioethics in Theory and Practice'.

## Aim

Our aim was to test empirically whether there is a difference in the ethical considerations or principles at stake between Danish physicians and Danish molecular biologists.

Research questions:

• Is there a difference in the ethical considerations or principles at stake between physicians and molecular biologists?

• Is there a difference in the ethical considerations or principles at stake between molecular biologists employed at a public university and molecular biologists employed in a private biopharmaceutical company?

## Study design and methods

The basic approach of the project was phenomenological hermeneutical. This approach was used both in the design of the interview guide and in the interpretation of data. However, to make the approach normative, the phenomenological hermeneutical method was combined with WRE as a decision procedure in the construction of the interview guide, as described in Ebbesen & Pedersen [7].

### The sample

This study is based on 12 interviews with physicians and molecular biologists (table 2). The type of sampling used was random purposeful (random selection to select limited numbers of cases from a larger purposeful sample). The sample size was determined in relation to data saturation. The decisive criterion for sample size is the point where variation ceases; saturation tends to occur when the number of interviews reaches around 15 ± 10 [33,34]. We observed that data saturation was beginning to appear after interviewing nine respondents (three respondents in each group). The inclusion criteria for this study were that the participants should have an academic degree in medical science or molecular biology and more than five years' working experience, so they have a thorough and profound knowledge of practice. Excluded from the study were people who did not meet the inclusion criteria, did not speak Danish, or had not been brought up in Denmark.

**Table 2:** Sample description

**Respondents **(number)	**Respondent group**	**Age **(years)	**Males **(number)	**Females **(number)
4	Oncology physicians working in a clinic at a public hospital	45–59	3	1
4	Molecular biologists employed at a public university working in a laboratory conducting basic research	31–57	2	2
4	Molecular biologists employed in a private biopharmaceutical company working in a laboratory conducting basic research	36–57	1	3

### Interview guide

The ethical reasoning of physicians and molecular biologists was explored by use of semi-structured interviews [34]. The interview guide used consists of 13 main questions (table 3), each containing a number of sub-questions (the sub-questions are not shown in the table). Each interview lasted for 1 hour and 5 minutes on average, and the interview was transcribed word-for-word in text form. There is a detailed description of the theory behind the interview questions in Ebbesen & Pedersen [7].

**Table 3:** Interview guide

The interview guide below was used in the present study of the ethical reasoning of physicians and molecular biologists. It consists of 13 main questions, each containing a number of sub-questions (the sub-questions are not presented here).
1. Please describe your background
2. Please describe your working day
3. What are the positive/satisfactory aspects of your job?
4. What are the negative/unsatisfactory aspects of your work?
5. In your profession, what makes a person qualified?
6. What are the perspectives of your research?
7. Have you ever been faced with difficult decisions about whether or not to participate in a research project? Or how to treat a patient?
8. Do you feel well-prepared to assess ethical problems about your participation in a research project? Or about what kind of treatment a patient should receive?
9. Presentation of an actual case:
In 2003, it was reported in *Science *that 2 out of 10 patients treated with retroviral mediated gene therapy against the immune system disease SCID-X1 developed leukaemia three years after the treatment. The gene therapy resulted in a functional immune system in 9 out of 10 patients, but 2 patients developed T cell leukaemia caused by insertional mutagenesis. What is your immediate assessment of this case?
10. Presentation of an actual case:
In 2002, the Danish newspaper *Information *reported how an Italian obstetrician had fertilized an infertile woman using the clone of a man. What is your immediate assessment of this case?
11. Presentation of bioethical principles:
Some bioethicists argue that four ethical principles have to be balanced when it comes to assessing bioethical cases: Respect for the patient's autonomy, an obligation to do good (beneficence), an obligation not to harm (nonmaleficence) and just and equal distribution of welfare services. How do you understand these concepts? Are these principles at stake in your practice?
Other bioethicists believe that the principle of respecting the patient's autonomy is too narrow to protect the human person, and that it should be supplemented with the principles of respect for the patient's dignity, vulnerability and integrity. How do you understand these concepts? Are these principles at stake in your practice?
12. Is the amount of time/resources available to you in your daily work sufficient to reflect on ethical issues?
13. Have you been involved in the implementation of concrete initiatives, projects or seminars about ethical issues in your profession?

Main questions in the interview guide used in the present study.

### Data analysis

The data were analysed using a phenomenological hermeneutical method for interpreting interview texts inspired by the theory of interpretation presented by Ricoeur as cited in Lindseth & Norberg [3] and Pedersen [35]. In the following, the three steps of data analysis are briefly described.

#### Naïve reading

The text is read several times in order to grasp its meaning as a whole. The interpreter tries to read the text with a phenomenological attitude, so as to be open enough to allow the text to speak to him/her. The naïve reading is regarded as a first conjecture and it has to be validated or invalidated by the subsequent structural analysis [3].

#### Structural analysis

According to Ricoeur, to understand a text is to follow its movement from what it says to what it talks about [3,35]. In the structural analysis, we move from what the text says to what it talks about, first describing units of meaning (what is said) and then identifying and formulating units of significance (what is talked about) and themes (table 4) [35].

**Table 4:** Example of structural analysis – the movement from what is said to what is talked about, first by describing units of meaning (what is said) and next by formulating units of significance (what is talked about) and themes.

**Respondent group**	**Units of meaning (What is said)**	**Units of significance (What is talked about)**	**Themes**
**Oncology physician working in a hospital clinic (OPC, Q1)**	... you meet a large number of extremely wonderful and brave people who by ill luck end up here due to serious illness ... most people deal with their fate extremely well ... they mobilise resources that people are not usually expected to possess. But most of them do ... It is an immense satisfaction for me ... when I have one of those tough outpatient departments, and I have seen 20 or 25 patients, and I can see that many of them have responded well to treatment, have recovered, are able to function and are happy and satisfied ... The patient's recovery is often preceded by a hard period of therapy during which he or she suffers a lot of pain and discomfort due to radiation therapy or an operation. And then the patient recovers. It is an immense satisfaction for me to witness that – there is nothing better, is there?	**Satisfaction by helping many brave people who mobilise reserves of energy to get through serious treatment**	**Beneficence**
**Molecular biologist employed in private biopharmaceutical company (MBP, Q2)**	It is a part of clinical development – to prove that it is safe ... not just for mice, but various animal models – it depends on the type of protein ... you have to prove that it does not produce cardiac problems or cancer ...	**Clinical trials to show that biopharmaceuticals are not potentially dangerous Animal models Cardiac problems Cancer**	**Nonmaleficence**
**Molecular biologist employed at the university (MBU, Q3)**	You must inform them of their options and then respect their decision.	**Inform patients and respect their decision**	**Respect for autonomy/Informed consent** • **Respect decision** • **External constraints**
**Oncology physician working in a hospital clinic (OPC, Q4)**	... try to determine what is wrong with the patient, what are our options, what are the patient's wishes, ideas, and then we have to reach some kind of mutual understanding, a frame of reference, and take it from there ... and how we can deal with this in respect of that.	**Medical prognosis Risk-benefit analysis Patient's wishes and ideas Mutual understanding Respect**	**Medical prognosis** • **Risk-benefit analysis** **Respect for autonomy/Informed consent** • **Patient's wishes and ideas** • **Mutual understanding** • **Respect**
**Molecular biologist employed in private biopharmaceutical company (MBP, Q5)**	... from a general perspective, we need a good reason for doing all the things we do. We are a PLC, so the biggest reason for us is the fact that we have to earn money for our shareholders, but we also need take into consideration the benefit of society, not just our own good, because all things are connected. If we start doing something unethical, then it will damage us in the long run ...	**Important for company to have ethical profile, since it pays off**	**Justice**
**Oncology physician working in a hospital clinic (OPC, Q6)**	Resources are scarce and the number of patients in need of radiotherapy is increasing ... you have so many patients and you want to be able to cure as many as possible from their cancer, which is, after all, the main problem. But what is the best way to do it so that the patients become most well-functioning afterwards, cosmetically and functionally?	**More patients than devices, how to manage resources most effectively**	**Justice** • **Just distribution**

First, the whole text is read and divided into units of meaning (what is said). These units of meaning can be part of a sentence, a sentence or a paragraph. Secondly, the units of meaning are reflected on in relation to the naïve understanding. Then the units of meaning are sorted and condensed and units of significance are formulated (what is talked about). Next, units of significance are condensed even more and themes are formulated. A theme is a thread of meaning that continues in several parts of the text. A theme identifies an essential meaning of lived experience; these themes are formulated as condensed descriptions and abstract concepts [3,35].

During the structural analysis, the text is viewed as objectively as possible by decontextualising the units of meaning from the text as a whole, so that the text parts are considered as independently as possible from their context in the text [3,35].

The themes are reflected on against the background of the naïve understanding to see whether the themes validate or invalidate the naïve understanding. If the structural analysis invalidates the naïve understanding, the whole text is read again and a new naïve understanding is formulated and checked by a new structural analysis. This process of comparing the naïve reading and the structural analysis is repeated until the naïve understanding is validated through the structural analysis [3,35].

#### Critical interpretation

The themes are reflected on in relation to the literature. The text is read again as a whole with the naïve understanding and the validated themes in mind and as open-mindedly as possible. However, according to Lindseth & Norberg [3], we interpret in relation to our pre-understanding and we cannot free ourselves from this pre-understanding. This is in line with Gadamer, who thinks that the hermeneutic mode of interpreting meaning is not without presuppositions, like the phenomenological description. The interpreter of a text cannot 'jump outside' the tradition of understanding he or she lives in [36,37]. The interpreter should, however, attempt to make such presuppositions or foreknowledge explicit [36]. Foreknowledge in this project includes the literature stated in the 'Reference' section in this article. According to Lindseth & Norberg [3], one can find literature that may be appropriate for helping to revise, widen and deepen our understanding of the text. This is where existing bioethical theory for data interpretation comes in. It can be used to structure the comprehensive understanding of the text, present alternative views and maybe revise the structure already made. However, since this was a phenomenological hermeneutical study, we did not force the literature perspective on the interview text, but let the literature illuminate the interview text and the interview text illuminate the literature [3,35].

## Results

From the structural analysis, a number of themes emerged, and these themes are explored in details below.

### The health of the patient in focus

Molecular biologists employed in a private biopharmaceutical company have the patient in focus when they plan research projects. They describe their research aims as gaining knowledge about cell, protein and gene functions in order to develop biopharmaceuticals to help patients. This means they have a specific effort of beneficence in mind. This is seen in MBP, Q7, below.

MBP, Q7:

... We are employed here to produce drugs for the benefit of the patients, so we have to see things in a broader perspective ... of course, that is not where your focus is when you are about to clone something and considering what restriction sites to apply. At that moment, the overall perspective is not the focus of your attention, but you have to keep it in the back of your mind as part of your daily activities in order for things to make sense in the end.

But although the individual researcher in the biopharmaceutical company has the beneficence of the patient in focus when planning new projects, the choice of the management of the company to start up a new project has to do with economics. This is seen in MBP, Q8, below.

MBP, Q8:

It requires a willingness to accept that you are not always able to finish your job completely, because sometimes you reach a conclusion where you have to admit that this part was not so good anyway – we may lack some answers, but we never get around to ... we cannot defend the continuation of the work, and therefore we close this activity now. You need to accept that you cannot complete all your work in detail, but the positive side is the fact that there are always exciting new projects to address and take further ... but, of course, you always have to weigh the financial aspects ... There needs to be an indication that this could potentially become a drug before it is even recognised as a project.

In contrast to molecular biologists employed in a private biotechnology company, molecular biologists employed at a public university do not have any specific effort of beneficence in mind; their research goal is to provide answers to interesting basic research questions so as to accumulate knowledge. They describe their research aims as gaining knowledge about fundamental cell, protein and gene functions. This is seen in MBU, Q9, below.

MBU, Q9:

... our overall goal is to understand how interferon works ... it is pure basic research.

Oncology physicians working in a clinic at a public hospital describe their research aims in a similar way to molecular biologists employed in a private biopharmaceutical company; they have a specific effort of beneficence in mind. Oncology physicians regard the goal of clinical trials as developing better treatment for patients. Their motivation is to help sick people. This is seen in quotation OPC, Q1, table 4. When deciding the inclusion criteria for a clinical trial, physicians firstly have the present patient in focus and consider how this experiment can benefit this patient. Secondly, the physician considers how this experiment can benefit future patients. This is seen in OPC, Q10, below.

OPC, Q10:

We have to give consideration to the patients first and foremost. If you are about to make an experiment, consideration for the patient comes first, but there are also all the future patients to consider. You have to constantly try to gain more insight, and perhaps you can kill two birds with one stone by offering a patient the chance to participate in an experiment.

Oncology physicians find that patients suffering from terminal illnesses want to participate in trials to benefit other patients. This is seen in OPC, Q11, below.

OPC, Q11:

The more ill you are, and the more hopeless your own situation is, the more ... So you try to attach some meaning to your situation, so that it can at least be to some use for others. Then I think you make a ... You try to find some sense in it and some ... Something positive is bound to come out of all this misery. If nothing else, greater knowledge on a collective basis.

### Risks and dangers

Molecular biologists employed in a private biopharmaceutical company or at a public university face minimal environmental and health risks regarding radioactivity and chemicals when conducting basic research. This is seen in quotation MBU, Q12, below. With regard to experiments in cell cultures, they believe that mammalian cells have a built-in safety margin against spreading to nature, since cells are sensitive and need to be treated well if they are not to die. Furthermore, molecular biologists experience very strict safety guidelines. This is seen in quotation MBP, Q13, below.

MBU, Q12:

It is minimal, but we do work with radioactivity, so that is one risk factor. But we use very small amounts ... a few chemicals that you should not eat directly ...

MBP, Q13:

The cells we use ... mammalian cells are generally very sensitive. If you do not treat them really, really well, they die ... So this is a margin of safety in itself ... I mean, against spreading to nature for instance ... and we also have systems to kill them before anything spreads to nature. As a precaution, we have very strict rules against pouring it down the sink. Everything needs to be ... sterilised. Everything is either autoclaved or we ... add something to the cell media and cell cultures that we use to kill the cells.

Molecular biologists employed in a private biopharmaceutical company carry out animal testing to investigate whether biopharmaceuticals may produce cardiac problems or cancer. This is seen in MBP, Q2, table 4 and in MBP, Q14, below.

MBP, Q14:

Clinical development is about finding out if whether or not this is an effective and safe treatment ... if it is not safe, then it is no use ... You cannot accept a treatment that makes people ill.

Molecular biologists employed at the university stress that toxicology tests are always performed in animal models and not in humans the first time. This is seen in MBU, Q15, below:

MBU, Q15:

Toxicology tests, for instance. You do not perform them on humans the first time. You always use some kind of test animal the first time.

To develop the most efficient cancer therapies with the fewest side effects, oncology physicians carry out clinical trials. They always consider how toxic the treatment is. When physicians decide whether or not to ask a patient to participate in a trial, the physician takes the situation of the patient into account. They consider whether the patient is suffering from a serious disease, whether the disease is curable or whether palliative care is needed. This is seen in quotations OPC, Q16–17, below.

OPC, Q16:

... how to offer a cancer treatment that would be both effective and cause as few side-effects as possible. That is the balance we need to strike. The balance between our effective remedies – that include surgery, radiation and chemotherapy – and the whole person who has be able to function afterwards.

OPC, Q17:

And of course, it must be viewed in the light of the seriousness of the situation. Are we dealing with potentially healthy patients or terminal ones? ... But in both cases you have to consider – even in the case of terminal patients: is it a real possibility? – and what kind of cost considerations are involved if they participate – how toxic is this?

According to molecular biologists employed at the university, ethical boards have to evaluate potential experiments or clinical trials in order to minimise the harm done to human subjects and patients. This is seen in quotation MBU, Q18, below:

MBU, Q18:

But somebody has to check that the professional skills and expertise are satisfactory before you ...otherwise, some smart Alec who makes an unconsidered choice ...

### Informed consent, external constraints, vulnerability and role reversal

In the study, we see examples of how molecular biologists find that informed consent can be influenced by external constraints. For instance, in MBU, Q3, table 4, a molecular biologist employed at the university says that patients must be informed of their options regarding treatment or trials and that their decision regarding these issues should be respected. This quotation indicates that informed consent should be respected without external constraints. However, in MBP, Q19, below, a molecular biologist employed in a private biopharmaceutical company stresses that very ill patients will accept any treatment, they will accept the risks involved, they are vulnerable and constrained by the circumstances to make a certain choice. MBU, Q20, presented below, illuminates the same issues and says that patients and human subjects should decide themselves, but that very ill patients are constrained by the circumstances to make a certain choice.

MBP, Q19:

... if you were a seriously ill or terminally ill patient, I think I would accept just about any treatment, because you would accept the risk involved.

MBU, Q20:

... people make their own choices; if you inform people of the risks, they must make the decision themselves. The problem is if they feel they are forced into it. Some may feel this way; it depends on the person.

Molecular biologists employed in a private biopharmaceutical company or at the university believe that if they were patients suffering from serious cancer, they would accept any treatment independent of side effects. They imagine themselves being in the situation of the patient (role reversal). This is seen in quotation MBU, Q21 and MBP, Q22, below.

MBU, Q21:

... often you try to put yourself in that person's shoes; if it was your child or if you were the patient, would you run that risk ...

MBP, Q22:

...if you tell a cancer patient: Do you want this treatment? – it will prolong your life by three months on average. If you were in that situation yourself, you would say: yes, give me this treatment. You could decide to live with various side-effects ...

### Respect for autonomy based on the patient's wishes and ideas, information and understanding

Oncology physicians believe that informed consent or refusal is based on the patient's wishes and ideas, information and understanding. For instance OPC, Q4, table 4, says that in dialogue with the patient, the physician performs a medical prognosis or risk-benefit analysis based on the patient's wishes and ideas, mutual understanding and respect. Furthermore, OPC, Q23, below, stresses that the physician has a positive obligation to adjust to the level of the patient when disclosing information to make sure that the patient understands. OPC, Q24, below, emphasises that the tasks of the physician are 1) to disclose information so that the patient can make informed consent, and 2) to respect this informed consent.

OPC, Q23:

... patients are very different and you must adjust to their level as best you can and try to work out what kind of language to speak and sense whether they have understood what you have told them, and maybe repeat it...

OPC, Q24:

My task is to ensure as well as possible that they know ... receive information on what we can offer and what options are available to them in their situation. And that the information is communicated in such a way that it forms the basis of their decision-making. If they then decide something else, then that is that.

### The principle of respect for autonomy does not apply

Oncology physicians think that there are situations, where the principle of respect for autonomy does not apply. OPC, Q25, below, describes such a situation.

OPC, Q25:

... when we have a protocol like that – there is the inclusion criteria ... the patient may meet all the criteria, but when I sit in front of the patient, I think to myself: this just will not work. This patient is in some sort of crisis or situation in which it is not fair to ask them to make this kind of decision. And then I can choose to say to myself that it is not fair. Then we give them the standard treatment ... once in a while I decide that they are not capable of making these decisions themselves. It is not fair to place the strain and stress of having to make such a decision on them – because it is a strain.

OPC, Q25 stresses that the physician's decision about treatment depends on the physical and psychological condition of the patient. Furthermore, the quotation tells us that the physician avoids asking the patient to make a decision if the patient is in a difficult situation, since it is not fair or just under these circumstances to place stress on the patient.

### Clinical trials in developing countries, fairness, limited resources, public funding and waiting lists

Molecular biologists employed in a private biopharmaceutical company have interdisciplinary working groups that discuss the ethical and legal issues of the use of human material and animal models. One reason the company prioritises ethical evaluation is because of the investors: if the company behaves unethically, they will be punished by the consumers and by the investors in the long run. This means it is important for the company to have an ethical profile, since it pays off. This is seen in MBP, Q5, table 4. The private biopharmaceutical company performs clinical trials in developing countries. To benefit the research subjects, they are assigned a lifetime of treatment. The company's motive in helping developing countries has to do with economics. This is seen in MBP, Q26, below.

MBP, Q26:

Generic drugs and placing drugs at the disposal of countries that cannot afford them. These are issues that management deals with, and there are guidelines ... on how to behave, if you make clinical experiments in a country that would not normally offer this kind of treatment. Then you should – well, not just should, but must – offer life-long treatment to the individuals involved, if it turns out that this clinical test is related to an effective treatment ... we have departments that are responsible for clinical experiments, and these are the kinds of things they deal with: Where can you carry out your clinical experiments, in which countries? And a number of things may speak in favour of the richest countries because then you will not be accused of ... exploitation. But there can also be good reasons for choosing less rich countries, because it gives them the opportunity to be included in treatment. And XX does it to some degree ... there are clinics established by XX in Africa, India and ... that are simply ... at XX's expense, and – you may say – compared to marketing budgets it is not a whole lot of money ... obviously, if you realise that it pays off. But at the same time it is a good thing.

Oncology physicians working in a clinic at a public hospital are dissatisfied with the management demands for efficiency. They think that budgets and demands for efficiency do not fit together and that the waiting lists are too long. The oncology physicians believe that they have to treat too many patients per day in the out-patients' clinic, since there is a limit to how many patients they can treat per day with empathy. This is seen in quotations OPC Q27–Q29, below.

OPC, Q27:

It is a demand from the management who ... it has been decided ... Budgets are decided and followed by demands and expectations of a specific number of patients to be attended to within the framework of the budget, and these ends do not meet. We quite often have waiting lists, and that is very unsatisfactory ...

OPC, Q28:

... You know there are already ten people in the waiting room and if you fall behind schedule, they will push you and ask you if it is not their turn? They are also nervous, because they are going to be tested. So it is all a race against time.

OPC, Q29:

.*.. it is dissatisfactory if the outpatient department programme is too tight ... if the production demand is too great. There is a limit to how many patients you can contain in your head in one day; to how much negative news you can give patients without becoming a tape recorder ... sort of a factory where things are weighed and measured that way.*

Oncology physicians find that resources are limited, they have too few instruments and too little equipment compared to the number of patients suffering from cancer, and they want to treat as many patients as possible in the best way. This is seen in OPC, Q6, table 4. However, the physician does not consider that resources are limited when deciding treatment for the individual patient. This is seen in OPC, Q30, below.

OPC, Q30:

I do not take into consideration that it will cost us 100.000 if we have to use Tascol again. I have not taken into consideration that it is too expensive. Not in relation to the individual patient, but of course, when we hold a meeting and the administrator informs us of the size of the current drugs budget, and that the expected payments deficit will be such and such if we initiate the new treatment, and we do not have the capacity or the staff to do it, and we do not have the means – or whatever – then you think about the financial aspects. But when you are facing the individual patient, you do not think too much about it.

Molecular biologists employed at a public university also find that resources are limited. They are stressed since they are under a constant pressure to apply for funding for research and the competition is hard. This is seen in MBU, Q31, below.

MBU, Q31:

...difficult to raise enough money to make ends meet. You are quite isolated, doing your own thing and raising money for it ... so basically we are competing for the same money ...publication is a prerequisite for being able to raise funds – so that is where the shoe pinches: to get your work financed to be able to continue...and if you need material for your test tubes, or equipment or the like, you have to find a way of getting it ... that is what puts me under the most stress, I would say ... excellent researchers do not work alone ...

## Discussion

In what follows, the findings of the structural analysis are interpreted and discussed in relation to the literature.

### Beneficence and nonmaleficence considerations

When interpreting the interview texts, we see similarities in the research aims of the group of molecular biologists employed in a private biopharmaceutical company and those of the oncology physicians working in a clinic. Both of these groups have the health of the patient in focus in their daily work. Oncology physicians regard the goal of clinical trials as developing better treatments with fewest side effects. Furthermore, oncology physicians find that patients suffering from terminal illnesses want to participate in trials to benefit other patients. Molecular biologists employed in a private biopharmaceutical company also have the patient in focus when they are planning research projects. They describe their research aims as gaining knowledge about cell, protein and gene functions in order to develop biopharmaceuticals to help patients. This means that the motivation of these groups is to help sick people by developing better treatment with fewer side effects. This reflects all three parts of the principle of beneficence of Beauchamp & Childress: 1) One ought to prevent and remove evil or harm, 2) One ought to do and promote good and 3) One ought to weigh and balance the possible goods against the possible harms of an action [16,17]. In contrast, molecular biologists employed at a public university do not have any specific effort of beneficence in mind; their research goal is to accumulate knowledge by providing answers to interesting basic research questions. They describe their research aims as gaining knowledge about fundamental cell, protein and gene functions.

The kinds of nonmaleficence considerations taken into account by the three groups of respondents differ significantly. The nonmaleficence considerations of molecular biologists employed at the university or in a private biopharmaceutical company are related to the environment, to the researchers' own health, to animal models, and only implicitly to human subjects. Molecular biologists face environmental and health risks with regard to radioactivity and chemicals, but they consider these as minimal. Furthermore, molecular biologists believe that the mammalian cells they use for experiments have a built-in safety margin against spreading to nature, since the cells are sensitive and need to be treated well if they are not to die. Molecular biologists employed in a private biopharmaceutical company perform tests on animal models to investigate whether biopharmaceuticals produce cardiac problems or cancer. Molecular biologists employed at the university state that such toxicology tests are always performed on animal models and not in humans the first time. They stress that the reason why ethical boards have to evaluate potential experiments or clinical trials is to minimise the harm done to research subjects and patients. From this we see, that the moral principle governing the treatment or non-treatment of patients is nonmaleficence. The obligation not to inflict harm is an obligation that co-travels with an obligation to test the drug in animal models before delivering the drug to human subjects or patients. This is also the reason ethical boards have to evaluate potential experiments or clinical trials. We have established procedures intended to minimise the harm done to patients and that reflect the principle of nonmaleficence and the first part of the principle of beneficence of Beauchamp & Childress.

The nonmaleficence considerations of oncology physicians have to do with patients or human subjects. Oncology physicians say that they perform clinical trials in order to develop the most efficient cancer therapies with the fewest side effects. For instance, they take into account how toxic chemotherapy is. When physicians decide whether or not to ask a patient to participate in a trial, the physician takes the situation of the patient into consideration: Does the patient suffer from a serious disease? Is the disease curable? Or does the patient need palliative care? As we see, physicians want to provide the maximum benefit but at the same time physicians do not want to introduce harm. Physicians consider the negative side effects of radiation and chemotherapy. They make judgements in each case as to whether or not the drug chosen is actually going to cause harm rather than hold out a substantial promise of benefit. The physician is concerned not only to benefit the patient but also not to harm the patient, which means that there is a beneficence consideration, a nonmaleficence consideration, and the balance between them. This reflects the principle of nonmaleficence and the three parts of the principle of beneficence of Beauchamp & Childress.

According to Beauchamp & Childress the evaluation of risk in relation to probable benefit is often labelled *risk-benefit analysis*. They say that the term *risk *refers to a possible future harm, where *harm *is defined as a setback to interests, particularly in life, health and welfare [16]. Statements of risk are both descriptive and evaluative. They are descriptive inasmuch as they state the probability that harmful events will occur, and they are evaluative inasmuch as they attach a value to the occurrence or prevention of these events [16]. Commonly in the field of biomedicine, the term *benefit *refers to something of positive value, such as life or health. The risk-benefit relationship may be conceived in terms of the ratio between the probability and magnitude of an anticipated benefit and the probability and magnitude of an anticipated harm. Use of the terms *risk *and *benefit *necessarily involves an evaluation. Values determine both what count as harms and benefits and how much weight particular harms and benefits have in the risk-benefit calculation [16]. The terms *harm *and *benefit*, defined as stated above, are ethically relevant concepts. Ethical obligations or principles about not inflicting harm (nonmaleficence) and promoting good (beneficence) are generally accepted [16]. According to Beauchamp & Childress, the balancing of the general ethical principles of nonmaleficence and beneficence is not symmetrical, since our obligation not to inflict evil or harm (nonmaleficence) is more stringent than our obligation to prevent and remove evil and harm or to do and promote good (beneficence) [16]. This was also shown in the present study, in all three groups of respondents: that the moral principle governing the treatment or non-treatment of patients (or participation in research) is nonmaleficence.

### Respect for autonomy as a negative obligation – informed consent, role reversal and vulnerability

In this study, we have seen how the two groups of molecular biologists employed in private biopharmaceutical company and at the university state that the informed consent of human subjects or patients can be influenced by external constraint. Molecular biologists say that patients must be informed of their options regarding treatment or trials and that their decision regarding these issues should be respected. This indicates that informed consent should be respected without external constraints. At the same time molecular biologists stress that very sick patients would accept any treatment, they would accept the risks involved, since they are vulnerable and constrained by the circumstances to make a certain choice. Following Beauchamp & Childress, this reflects the principle of respect for autonomy as a negative obligation which says that autonomous actions should not be subjected to controlling constraints by others [16].

### Respect for autonomy as a positive obligation – information, understanding and the patient's wishes and ideas

Like molecular biologists, oncology physicians experience the principle of respect for autonomy as a negative obligation, since they see informed consent of the patient as an obligation that should be respected without external constraint, i.e. the physician must respect the decision of the patient even when it is not the one he/she has recommended.

However, in contrast to molecular biologists, oncology physicians also experience the principle of respect for autonomy as a positive obligation. The principle of respect for autonomy as a positive obligation requires respectful treatment in disclosing information, probing for and ensuring understanding and voluntariness, and fostering autonomous decision-making [16]. This is seen in the way oncology physicians believe that they have a positive obligation to adjust to the level of the patient when providing this information to make sure that the patient understands. Furthermore, in dialogue with the patient, they perform a medical prognosis or risk-benefit analysis based on the patient's wishes and ideas, with mutual understanding and respect.

### The principle of respect for autonomy does not apply – the patient is not competent

Oncology physicians believe that there are situations, where the principle of respect for autonomy does not apply. This study shows that physicians avoid asking the patient to make a decision if the patient is in a difficult situation, since it is not fair or just under these circumstances to place stress on the patient. This means that the physician's decision about treatment depends on the physical and psychological condition of the patient. Following Beauchamp & Childress, this describes situations in which the principle of respect for autonomy does not apply because of the physical and psychological condition of the patient. The patient is not competent, i.e. the patient is not able to make autonomous decisions. Beauchamp & Childress define competence to decide about treatment or about participation in research the following way: "Patients or subjects are competent to make a decision if they have the capacity to understand the material information, to make a judgement about the information in light of their values, to intend a certain outcome, and to communicate freely their wishes to care givers or investigators" [16]. According to Beauchamp & Childress, when the principle of respect for autonomy does not apply, the principles of beneficence and nonmaleficence step in [16].

Situations where the principle of respect for autonomy does not apply can also be considered as justice situations: it is not fair or just under the circumstances to ask the patient in that specific situation to participate in research. Basically, it is about inclusion criteria. But the case can also be looked at from the point of view of keeping the well-being of the patient in focus. According to the physician, it is too great a burden to put on the patient in that specific situation to ask him/her to participate in research. Following Beauchamp & Childress' theory, the physician balances the principles of beneficence and nonmaleficence.

### Justice – fairness, waiting lists and limited resources

This study shows that the private biopharmaceutical company prioritises ethical evaluation of research because of the investors. If the company behaves unethically, they will be punished by the consumers and by the investors in the long run. So it is important for the company to have an ethical profile, since it pays off. For instance, the study shows that the private biopharmaceutical company performs clinical trials in developing countries and in order to be fair and to benefit the human subjects, they are assigned a lifetime of treatment. The motive of the company to help people living in developing countries has to do with economics. This is in line with the fact that although the individual researcher in the biopharmaceutical company has the beneficence of the patient in focus when planning new projects, the choice of the management of the company to start up a new project has to do with economics. This means that the motive of the private company helping people in developing countries and in carrying out ethical evaluation has to do with economics, but all things considered it has good consequences. From this we can see that the principle of justice proposed by the company has utilitarian characteristics. Utilitarianism is a theory that prescribes the quantitative maximisation of good consequences for a population. It is a form of consequentialism. The good to be maximised is usually happiness, pleasure, or preference satisfaction. These consequences generally have something to do with the welfare of people.

Oncology physicians working in a clinic at a public hospital are dissatisfied with management demands for efficiency, that budgets and demands for efficiency do not fit together, and that the waiting lists are too long. Oncology physicians believe that they have too many patients to treat per day in the out-patients' clinic, since there is a limit to how many patients they can treat per day with empathy. Furthermore, oncology physicians find that resources are limited, they have too few instruments and too little equipment compared to the number of patients suffering from cancer and they want to treat as many patients as possible in the best way. However, the physician does not consider that resources are limited when deciding treatment for the individual patient. When deciding the inclusion criteria for a clinical trial, physicians firstly have the present patient in focus and consider how this experiment can benefit this patient. Secondly, the physician considers how this experiment can benefit future patients. From this we see that the overall concept of justice proposed by oncology physicians has utilitarian characteristics, since they want to treat as many patients as possible in the best way with the resources allocated. However, we also see that the individual patient is not sacrificed for the benefit of future patients.

Just like oncology physicians, molecular biologists employed at a public university find that resources are limited, they think that a lot of time is spent on paperwork, administration and fund-raising. They feel stressed since they are under a constant pressure to apply for external funding for research and the competition is hard. So molecular biologists employed at a public university find that public funding for research is limited and that they are forced to apply for external funding which is time and energy-consuming. This means that they reflect about allocation, but they do not propose a specific theory of allocation or justice.

As indicated above, the concept of justice proposed by the private biopharmaceutical company and by oncology physicians has utilitarian characteristics. However, the respondents do not touch upon the principle of justice reflected in the way health care resources are allocated in Denmark as a whole. Beauchamp & Childress do describe how the systems of health care financing and delivery in Scandinavian countries are organised. They state that the system in these countries primarily looks to egalitarian justice, with utility as a secondary consideration [16]. Egalitarian theories emphasise "equal access to the goods in life that every rational person values (often invoking material criteria of need and equality)" [16]. Beauchamp & Childress [16] describe the Scandinavian systems as unified national systems, which in principle cover all citizens without reference to age, health status, medical condition or employment status. The justification for the system is that only government can provide universal coverage and cover increases in health care expenditures. Every person gets national health care, pays no charges for services, is free to choose a provider, and is eligible to receive the services covered, which include long-term and chronic care services. Physicians are free to work on a salaried basis or to practise privately. Regional boards establish fees and no physician is allowed to charge the government more than the amount set by the boards. Among the controversial features of this system are the elimination or near elimination of competitive aspects in the financing system and the resulting disutility.

Beauchamp & Childress propose a principle of justice that includes both utilitarian and egalitarian standards. They write: "In particular, we have proposed that society recognize an enforceable right to a decent minimum of health care within a framework for allocation that incorporates both utilitarian and egalitarian standards" [16]. This study therefore indicates that the principle of justice proposed by Beauchamp & Childress is applied at the level of individual physicians, in the biopharmaceutical company, and according to Beauchamp & Childress also at the level of the system of health care allocation as a whole in Denmark.

## Conclusion

This empirical study shows that the principle of beneficence proposed by Beauchamp & Childress, which requires the taking of action to help – by preventing harm, removing harm and promoting good [16] – is at stake in the daily work of oncology physicians employed in a clinic and in the daily work of molecular biologists employed in a private biopharmaceutical company, since the motivation of both these groups is to help sick people.

Furthermore, according to this study, the principle of nonmaleficence of Beauchamp & Childress is at stake in the daily work of each of the three groups of respondents. However, there are differences with regard to the kinds of nonmaleficence considerations in the different groups. The nonmaleficence considerations of the two groups of molecular biologists are related to the environment, to the researchers' own health, to animal models, and only implicitly to human subjects. In contrast, the nonmaleficence considerations of oncology physicians have to do with patients or human subjects.

The fact that molecular biologists investigate DNA and cells in cultures, whereas physicians work with human beings in a doctor-patient relationship might have the effect that molecular biologists and oncology physicians perceive or experience the principle of respect for the autonomy of patients or human subjects in different ways. The negative obligation of Beauchamp & Childress' principle of respect for autonomy, which says that autonomous actions should not be subjected to controlling constraints by others, is reflected in the experience of both oncology physicians and molecular biologists in their daily work. But the positive obligation of the principle of Beauchamp & Childress, which requires respectful treatment in disclosing information, probing for and ensuring understanding and voluntariness, and fostering autonomous decision-making is only reflected in the experience of oncology physicians and not of molecular biologists. In dialogue with the patient, physicians perform a medical prognosis based on the patient's wishes and ideas, with mutual understanding and respect.

There are situations in which the patient is not competent and the principle of respect for autonomy does not apply. According to Rendtorff & Kemp [30], this is where other supplementary principles must be taken into account such as respect for dignity, integrity and vulnerability to protect the human person. Rendtorff & Kemp [30] accuse the theory of Beauchamp & Childress of having too narrow a concept of the human person by regarding the principle of respect for autonomy as the primary principle. However, according to Beauchamp & Childress, the principles of beneficence and nonmaleficence step in if the patient is not competent [16]. Actually, this study shows that the principles of beneficence and nonmaleficence actually do step in when the patient is not competent and the principle of respect for autonomy does not apply.

From the study we see, that molecular biologists employed at a public university do not propose a specific theory of justice. The principle of justice proposed by oncology physicians and by the private biopharmaceutical company has utilitarian characteristics. Furthermore, the health care system in Denmark primarily looks to egalitarian justice, with utility as a secondary consideration [16]. The principle of justice proposed by Beauchamp & Childress includes both utilitarian and egalitarian standards. This study therefore indicates that the principle of justice of Beauchamp & Childress is applied both at the level of the individual physician and the biopharmaceutical company and, according to Beauchamp & Childress, at the level of the system of health care allocation as a whole in Denmark.

In conclusion, this study shows that each of the four bioethical principles of Beauchamp & Childress is reflected in the daily work of oncology physicians and molecular biologists and thereby indicates that the principles are applicable to the Danish biomedical setting. In this study we have not simply systematically described the ethical reasoning of oncology physicians and molecular biologists. By taking an approach to bioethics in which we combine elements from theory and practice, where empirical research is integrated into the formulation of normative ethical principles, we believe that the conclusions of this study might provide health care professionals and biomedical researchers with normative principles that are concordant with their biomedical practice and which have normative implications.

## Future perspectives

Beauchamp & Childress claim that their bioethical principles are part of a cross-cultural common morality [16], but some of their critics state that the principle-based theory is developed from a shared American morality and mirrors certain aspects of American society, and that it might for this reason alone be non-transferable to other contexts and other societies [24]. Nonetheless, the findings of this article indicate that the principles of respect for autonomy, beneficence, nonmaleficence and justice are transferable to Danish biomedical practice. Future perspectives of this study are to investigate biomedical practice in several different cultures, for instance Asian, American and Southern European cultures, to test whether Beauchamp & Childress' principles are cross-cultural and thus have a universal perspective.

## Appendix

Note 1: Following Tom L. Beauchamp & James F. Childress, we understand the term bioethical principles as "general norms that leave considerable room for judgment in many cases. They do not act as precise action guides that inform us in each circumstance how to act in the way that more detailed rules and judgments do" [16].

Note 2: The notion of 'coherence' is not well defined. Philosophers agree that coherence is not only characterised by consistency [38,39]. According to the Danish philosopher, Klemens Kappel [39], coherence is characterised by consistency, systematicity (a belief set should contain explanatory relations), generality (a belief set should contain general beliefs that cover a larger area rather than a smaller one), and simplicity (general explanatory beliefs should be few and simple rather than many and complex).

Note 3: Our note. According to Rawls, a competent judge possesses the following characteristics: Intelligence and knowledge, intellectual virtues of reasonableness, an open mind, and sympathetic knowledge of those human interests which give rise to the need to make a moral decision [11].

## Abbreviations

(XX): Indicating the name of the company.

(OPC, Q1): Oncology Physician working in a Clinic at a public hospital, Quotation 1.

(MBP, Q2): Molecular Biologist employed in a Private biopharmaceutical company, Quotation 2.

(MBU, Q3): Molecular Biologist employed at a public University, Quotation 3.

(PLC): Public Limited Company (quoted on the stock exchange).

## Competing interests

The authors declare that they have no competing interests.

## Authors' contributions

Mette Ebbesen and Birthe D. Pedersen were responsible for the entire manuscript.
